# Lord Salisbury's Article

**Published:** 1886-12-11

**Authors:** 


### "THE HOSPITAL" AND "THE GLOBE."

### LORD SALISBURY'S ARTICLE.

It was with some pain that we last week published a statement with reference to the publication of Lord Salisbury's article in the fifth edition of *The Globe* of Wednesday, the 24th ult. We then explained that we were unable to give details because the matter might come before the Courts.

The matter has since been submitted to counsel, the whole of the evidence has been thoroughly sifted, and we are advised that "the course adopted by the proprietors of *The Globe* is a good ground for an action for damages against them."

In ordinary circumstances a writ would, of course, be immediately issued to recover compensation for the injury done to this Journal. The mission of The Hospital is, however, wholly peaceful and philanthropic, and its conductors prefer to take the more consistent course of standing by their principles, by refraining from retaliating on *The Globe*, notwithstanding the aggravating circumstance that not one word of apology or explanation has yet been published by the conductors of that paper.

The publication of the following document, prepared by the solicitor and counsel of this Journal, will afford the Press and the public full facilities for passing judgment on a transaction which we prefer not to characterise.

